# Age-dependent changes in the transverse carpal ligament and median nerve: a cadaveric histological and biomechanical study

**DOI:** 10.7717/peerj.20878

**Published:** 2026-02-24

**Authors:** Apichaya Niyomchan, Akkradate Siriphorn, Kanet Kathinted, Benjaporn Pamornpol

**Affiliations:** 1 Department of Anatomy, Faculty of Medicine Siriraj Hospital, Mahidol University, Bangkok, Thailand; 2 Department of Physical Therapy, Faculty of Allied Health Sciences, Chulalongkorn University, Bangkok, Thailand

**Keywords:** Aging, Transverse carpal ligament, Median nerve, Carpal tunnel, Mechanical property

## Abstract

**Background:**

Age-related alterations in the transverse carpal ligament (TCL) and the median nerve are thought to increase susceptibility to carpal tunnel syndrome in older individuals. This study aimed to investigate the mechanical properties of the TCL and histological changes in both the TCL and the median nerve in cadavers across a wide age range.

**Methods:**

Fifty formalin-embalmed cadavers (40–93 years old) were studied, yielding 100 TCL specimens. A digital palpation device (MyotonPRO) was used *in situ* to measure TCL dynamic stiffness, elasticity (logarithmic decrement), mechanical stress relaxation time, and creep at proximal, middle, and distal regions. After testing, each TCL and corresponding median nerve were excised. Masson’s trichrome staining and scanning electron microscopy were performed to assess collagen fiber organization, fibroblast density, and nerve structure. Pearson’s correlation was used to determine associations between TCL properties and age.

**Results:**

An age-dependent increase in TCL stiffness was observed, alongside decreased elasticity, relaxation time, and creep. Histological analyses revealed reduced fibroblast density, disorganized collagen fibers with large clefts between bundles, and increased fine collagen meshwork in interfascicular matrix in older specimens. Furthermore, thinning of connective tissue layers surrounding the median nerve and diminished myelin sheaths were noted with advancing age.

**Conclusion:**

This cadaveric study reveals that the TCL stiffens and becomes structurally disorganized with advancing age, paralleled by degenerative changes in the median nerve. Such age-related alterations may predispose elderly individuals to a higher risk of carpal tunnel syndrome, underscoring the need for targeted preventive and therapeutic strategies.

## Introduction

The global population is aging rapidly, with the number of individuals aged 60 years or older continuing to rise. According to the World Health Organization, the worldwide older population is expected to increase from one billion in 2020 to 1.4 billion in 2030 and to 2.1 billion by 2050 (World Health Organization, 2024). Understanding the aging processes that affect the health and well-being of the elderly is therefore of paramount importance.

Age-related changes have been documented in virtually all organs and systems, including the musculoskeletal system—encompassing skeletal muscle, tendons, ligaments, bones, and articular cartilage (Frontera, 2017). Ligaments, which connect bone to bone *via* fibrous connective tissue bands, undergo alterations in cellular function, extracellular matrix composition, and mechanical properties as individuals grow older (Ellis & Weiss, 2015). Fibroblasts—the primary cells in ligaments—experience phenotypic and functional changes, while collagen fibrils in the extracellular matrix can become disorganized and less flexible. These modifications heighten susceptibility to injuries or chronic pain and may restrict joint motion over time (Peters et al., 2022).

Among the ligaments of the wrist, the transverse carpal ligament (TCL)—also known as the flexor retinaculum of the wrist—has been the subject of extensive study due to its anatomic significance. Quantitative examinations have shown that TCL thickness can vary considerably, from approximately 0.8 to 6.0 mm, depending on the location along the proximal-distal axis of the carpal tunnel (Cobb et al., 1993; Pacek et al., 2010; Tanzer, 1959). For instance, one study reported that the proximal samples are thicker and have greater elastic moduli than distal samples, while radial and ulnar segments tend to be thicker than middle portions (Holmes et al., 2012). Histologically, the TCL consists mainly of type I and III collagen fibers arranged predominantly in the transverse orientation, with additional oblique patterns in the pisiform-trapezium and scaphoid-hamate regions, and some longitudinal fibers (Isogai et al., 2002; Prantil et al., 2012). Neural elements, such as free nerve endings (nociceptors) and Pacinian corpuscles (mechanoreceptors), have also been identified within and around the TCL (Mashoof et al., 2001).

Advancing age appears to affect not only the TCL’s structure but also its biomechanical integrity. Previous studies have shown that collagen synthesis rates in ligaments tend to decline with age, leading to a decrease in reducible collagen cross-links and an increase in non-reducible cross-links (Amiel et al., 1991). Although some investigations have revealed that stiffness and ultimate load in human ligaments (*e.g*., anterior cruciate ligament of the knee) may decrease with age (Robinson, Bull & Amis, 2005; Wilson et al., 2012; Woo et al., 1991), others report different outcomes depending on the specific ligament type and function. Collectively, these findings underscore that aging ligaments often become more susceptible to various forms of damage.

In addition to ligamentous changes, aging is associated with progressive structural and functional alterations of the median nerve. Previous studies have reported age-related reductions in nerve conduction velocity, axonal loss, thinning and fragmentation of myelin sheaths, and changes in the amount of nerve fibers and connective tissue elements within peripheral nerves. These degenerative changes may impair nerve resilience and exacerbate susceptibility to compression neuropathies, such as carpal tunnel syndrome (CTS), in older individuals (Sladjana, Ivan & Bratislav, 2008; Pannese, 2011; Li et al., 2015). CTS results from compression or entrapment of the median nerve at the wrist, beneath the TCL. Although more common in working-age adults—especially women—CTS in the elderly is rising in prevalence, reaching 22.2% among those over 55 years of age (Bland & Rudolfer, 2003; Cazares-Manríquez et al., 2020). Older patients often experience more severe symptoms, such as pain, numbness, and sensory disturbances in the thumb, index, and middle fingers (Dabbagh et al., 2020; Ko et al., 2018) and tend to be less satisfied with both conservative and surgical treatments (Fung, Tang & Fung, 2015; Vyšata et al., 2014). One study found that bowing of the TCL correlates strongly with age, suggesting that the ligament’s morphological changes may contribute to CTS etiology (Altinok & Karakas, 2004). Additionally, reduced collagen flexibility and increased soft-tissue volume in the carpal tunnel among the elderly could exacerbate median nerve compression (Duncan, Bhate & Mustaly, 2017).

The TCL itself serves multiple critical functions in the wrist, including providing a stable transverse arch for the carpal bones, anchoring the thenar and hypothenar muscles, and acting as a pulley for the flexor tendons (Li et al., 2014; Mhanna, Marquardt & Li, 2016). Because of its pivotal biomechanical role, age-induced changes in TCL thickness, mechanical properties, and overall tissue quality may help explain the elevated incidence and severity of CTS in older populations (Fung, Tang & Fung, 2015; Stecco et al., 2010). Although numerous studies have independently investigated age-related changes in the TCL or pathological alterations of the median nerve, the combined effects of aging on both structures within the carpal tunnel remain poorly characterized. In particular, few studies have integrated biomechanical assessment of the TCL with detailed histological evaluation of the median nerve across different ages.

Accordingly, this study aimed to investigate the effects of aging on both the mechanical and structural properties of the TCL, as well as to explore corresponding changes in the median nerve lying immediately deep to the ligament. Specifically, we sought to (1) evaluate the mechanical properties of the TCL, (2) examine morphological and histological changes in the TCL, and (3) assess age-related alterations of the median nerve beneath the TCL. The insights gained from this research may be beneficial in preventing and mitigating the risk of developing CTS among the elderly.

## Materials and Methods

### Specimen

One hundred TCL specimens—50 from right hands and 50 from left hands—were obtained from 12-month formalin-embalmed cadavers (21 males, 29 females), aged 40–93 years, without lesions or pathologies in the hands or wrists. Information regarding systemic medical conditions such as diabetes, amyloidosis, or neurodegenerative diseases was not available due to limitations of cadaveric donation records. However, all specimens were grossly examined to exclude visible pathology or injury of the hand and wrist. These cadavers were provided by the Department of Anatomy, Faculty of Medicine Siriraj Hospital, Mahidol University, between May, 2022 and December, 2024. Written informed consent was personally signed by all donors for educational and medical research purposes. All cadaveric data were fully anonymized prior to being accessed for this study. The study protocol was approved by the Siriraj Institutional Review Board (SIRB), Faculty of Medicine Siriraj Hospital, Mahidol University under an SIRB exempt research (SIRB No. 298/2565 (Exemption)).

### Mechanical test

After removing the skin, subcutaneous fat, and fascia from the wrist and palm of each cadaveric forearm, the TCL was exposed. A MyotonPRO device (Myoton AS, Estonia) was then employed *in situ* to measure four non-invasive parameters detailing tissue properties (Prantil et al., 2012). Specifically, stiffness (dynamic stiffness (N/m)) indicates the tissue’s resistance to an external force that changes its shape. Elasticity (logarithmic decrement) represents how quickly oscillations diminish within the tissue, where higher values indicate lower elasticity. Relaxation time (mechanical stress relaxation time (ms)) is the duration for the tissue to return to its original shape once the external force is removed, with lower values indicating more rapid elastic recoil. Finally, creep (ratio of relaxation and deformation time) describes the gradual deformation of the tissue under a constant load over time; lower creep values denote greater resistance to shape change. Measurements were obtained by positioning the MyotonPRO probe perpendicularly at three distinct sites on the TCL—proximal, central, and distal—all located above the median nerve. The proximal and distal sites corresponded to the respective edges of the TCL, while the central site lay midway between them. Each location was measured three times, and the average of these repeated measurements was used for data analysis.

### Histological study

For histological analysis, specimens from the youngest (<60 years, *n* = 7) and oldest (≥80 years, *n* = 17) age groups were selected to emphasize age-related differences. This approach was chosen to maximize contrast between extreme age groups. Following the biomechanical tests, each TCL and carpal tunnel contents were excised and immersed in 10% formalin for 24 h at room temperature. Subsequently, each ligament was divided into proximal, middle, and distal segments, rinsed in running tap water overnight, and dehydrated in ascending concentrations of ethanol (70%, 85%, 95% I, 95% II, absolute alcohol I, and absolute alcohol II), each step lasting 3 h. The specimens were then cleared in two xylene baths (3 h each), infiltrated with molten paraffin in three successive 3-h steps, embedded in paraffin blocks, and sectioned at 6-µm thickness using a rotary microtome (HistoCore BIOCUT; Leica Biosystems, Nussloch, Germany).

Masson’s trichrome staining was employed to visualize the TCL’s cellular and extracellular matrix components. In brief, deparaffinization was carried out by immersing the sections in xylene I and xylene II for 30 min each, followed by rehydration in a graded ethanol series (absolute alcohol I, absolute alcohol II, 95%, and 80%) for 5 min per step. Slides were then rinsed in running tap water and transferred to Bouin’s fixative at 56 °C for 1 h, promoting optimal staining of collagen. After cooling and thorough washing to remove the yellow color, slides were stained with Weigert’s iron hematoxylin solution for approximately 10 min, which rendered cell nuclei a dark purple/black. They were next treated with Biebrich scarlet-acid fuchsin to stain cytoplasm and muscle fibers red. A phosphomolybdic acid-phosphotungstic acid solution was subsequently applied to enhance collagen contrast, followed by immersion in aniline blue solution, which specifically highlights collagen in shades of blue. The stained slides were quickly rinsed in distilled water, immersed in 1% acetic water for differentiation, and then dehydrated in ascending grades of ethanol (95%, absolute I, absolute II), cleared in multiple xylene baths, and mounted under a coverslip with permanent mounting media. All stained sections were analyzed under an Olympus Bx43 microscope equipped with a DP73 digital camera (Olympus Optical Co., Ltd., Tokyo, Japan) to examine collagen fiber organization, fibroblast distribution, and overall tissue architecture.

Fibroblast density was quantified by counting cell numbers per 40,000 μm^2^ area (40X magnification field) in 60 images per age group, focusing on the youngest (<60 years) and oldest (≥80 years) groups for comparison.

Moreover, special attention was given to the connective tissue layers surrounding the nerve (mesoneurium, epineurium, perineurium, and endoneurium), as well as to the myelinated fibers and axons within the nerve fascicles.

### Scanning electron microscopy

For ultrastructural analysis, a 1 × 1 cm^2^ segment was obtained from the central region of each TCL (above the median nerve), ensuring minimal inclusion of adjacent fascial tissues. These tissue blocks were immersed in 2.5% glutaraldehyde for at least 1–2 h at room temperature to stabilize cellular and extracellular components. After initial fixation, they were rinsed twice (10 min per rinse) in 0.1 M phosphate-buffered saline (PBS, pH 7.4) to remove residual fixative, followed by three washes (10 min each) in distilled water. The samples were then post-fixed in 1% osmium tetroxide (in 0.1 M PBS, pH 7.4) for 1 h, enhancing membrane contrast and preserving ultrastructural details.

Post-fixation was followed by a graded ethanol dehydration series at 30-min intervals in 70%, 80%, 90%, 95% I, 95% II, 100% I, and 100% II ethanol. Complete dehydration was achieved by critical point drying in liquid CO_2_, which prevents structural collapse of delicate collagen fibrils. Dried specimens were subsequently mounted on metal stubs using double-sided conductive tape and coated with a thin layer of gold in a sputter coater. The prepared samples were examined under a LEO 1450VP scanning electron microscope (Carl Zeiss, Oberkochen, Germany) at an accelerating voltage of 15 kV, with images acquired at low to high magnifications to visualize collagen fiber organization, interfascicular matrix distribution, and any microstructural changes within the TCL.

### Statistical analysis

All biomechanical data (dynamic stiffness, logarithmic decrement, mechanical stress relaxation time, and creep) were recorded as mean ± standard error of the mean (SEM). Statistical analyses were conducted using GraphPad Prism version 5.0 (GraphPad Software, Inc., San Diego, CA, USA). One-way analysis of variance (ANOVA) was used to compare biomechanical properties among age groups because the independent variable (age group) was categorical and the dependent variables were continuous. Pearson’s correlation analysis was used to assess linear relationships between age and individual biomechanical parameters. One-tailed tests were applied based on *a priori* hypotheses that aging would be associated with increased stiffness and reduced elasticity-related properties. Correlation coefficients (r) were classified as negligible (0.00–0.10), weak (0.10–0.39), moderate (0.40–0.69), strong (0.70–0.89), or very strong (0.90–1.00) (Schober, Boer & Schwarte, 2018). Student’s *t*-test was used to compare fibroblast cell numbers between <60 and ≥80 age groups. A *p*-value less than 0.05 indicated statistical significance.

## Results

### General cadaveric characteristics

Table 1 summarizes the demographic data for the 50 cadavers (100 TCLs) included in this study. The mean cadaveric age was 73.24 ± 1.25 years, with an overall age range of 40–93 years. When stratified by age groups, the mean ages were 51.14 ± 1.56 years for those under 60, 64.73 ± 0.74 for those aged 60–69, 75.40 ± 0.50 for those aged 70–79, and 85.94 ± 0.67 for those aged 80 and older. Of the total cadavers, 42% (*n* = 21) were male and 58% (*n* = 29) were female.

**Table 1:** The demographic information of cadavers.

Characteristic	Age (years) Mean ± SEM	Age (years) Minimum-Maximum
**Gender (*n*, %)**		
• Male (21, 42%)	74.19 ± 1.60	51–93
• Female (29, 58%)	72.55 ± 1.82	40–92
**Age average**	73.24 ± 1.25	40–93
**Subject by age groups (*n*, %)**		
• <60 (7, 14%)	51.14 ± 1.56	40–59
• 60–69 (11, 22%)	64.73 ± 0.74	60–69
• 70–79 (15, 30%)	75.40 ± 0.50	71–79
• ≥80 (17, 34%)	85.94 ± 0.67	80–93

### Mechanical property of the TCL

The TCL exhibited notable age-related changes in four key mechanical parameters: dynamic stiffness, logarithmic decrement, mechanical stress relaxation time, and creep (Table 2). Although measurements were obtained at three anatomical regions, the present analysis focused on age-related differences using averaged values rather than regional comparisons. Dynamic stiffness values in all regions—proximal, middle, and distal—rose significantly with advancing age, with a particularly large jump observed in the ≥80 age group, as indicated by the superscripts a, b, and c in Table 2 (one-way analysis of variance (ANOVA) with Tukey’s honestly significant difference *post-hoc* test).

**Table 2:** Dynamic stiffness, logarithmic decrement, mechanical stress relaxation time, and creep of transverse carpal ligament based on age groups.

Age groups	Parts of transverse carpal ligament			
	Proximal	Middle	Distal	Average (three parts) ± SEM
**Dynamic stiffness (N/m)**				
**<60**	1,401.98	1,588.88	1,549.48	1,513.44 ± 31.55
**60–69**	1,639.38	1,671.86	1,603.05	1,638.10 ± 97.06
**70–79**	1,844.92	2,006.19	1,992.89	1,948.00 ± 122.50^a^
**≥80**	2,808.45	2,825.66	2,706.94	2,780.35 ± 46.05^a^^,^^b^^,^^c^
**Logarithmic decrement**				
**<60**	1.73	1.75	1.69	1.73 ± 0.06
**60–69**	1.73	1.74	1.64	1.70 ± 0.05
**70–79**	1.80	2.04	1.74	1.86 ± 0.10
**≥80**	1.87	2.07	1.76	1.90 ± 0.02
**Mechanical stress relaxation time (ms)**				
**<60**	7.94	8.70	8.53	8.39 ± 0.19
**60–69**	7.99	8.02	7.71	7.91 ± 0.37
**70–79**	5.87	6.28	6.30	6.15 ± 0.45^a^^,^^b^
**≥80**	3.42	3.18	3.51	3.37 ± 0.17^a^^,^^b^^,^^c^
**Creep (ratio of deformation and relaxation time)**				
**<60**	0.99	1.16	1.10	1.09 ± 0.03
**60–69**	1.05	1.04	0.97	1.02 ± 0.05
**70–79**	0.71	0.91	0.81	0.81 ± 0.06^a^^,^^b^
**≥80**	0.47	0.45	0.46	0.46 ± 0.02^a^^,^^b^^,^^c^

**Notes:**

One-way ANOVA with Tukey HSD for *post-hoc* test.

a significant difference as compared to <60 group.

b significant difference as compared to 60–69 group.

c significant difference as compared to 70–79 group.

**Figure 1:**
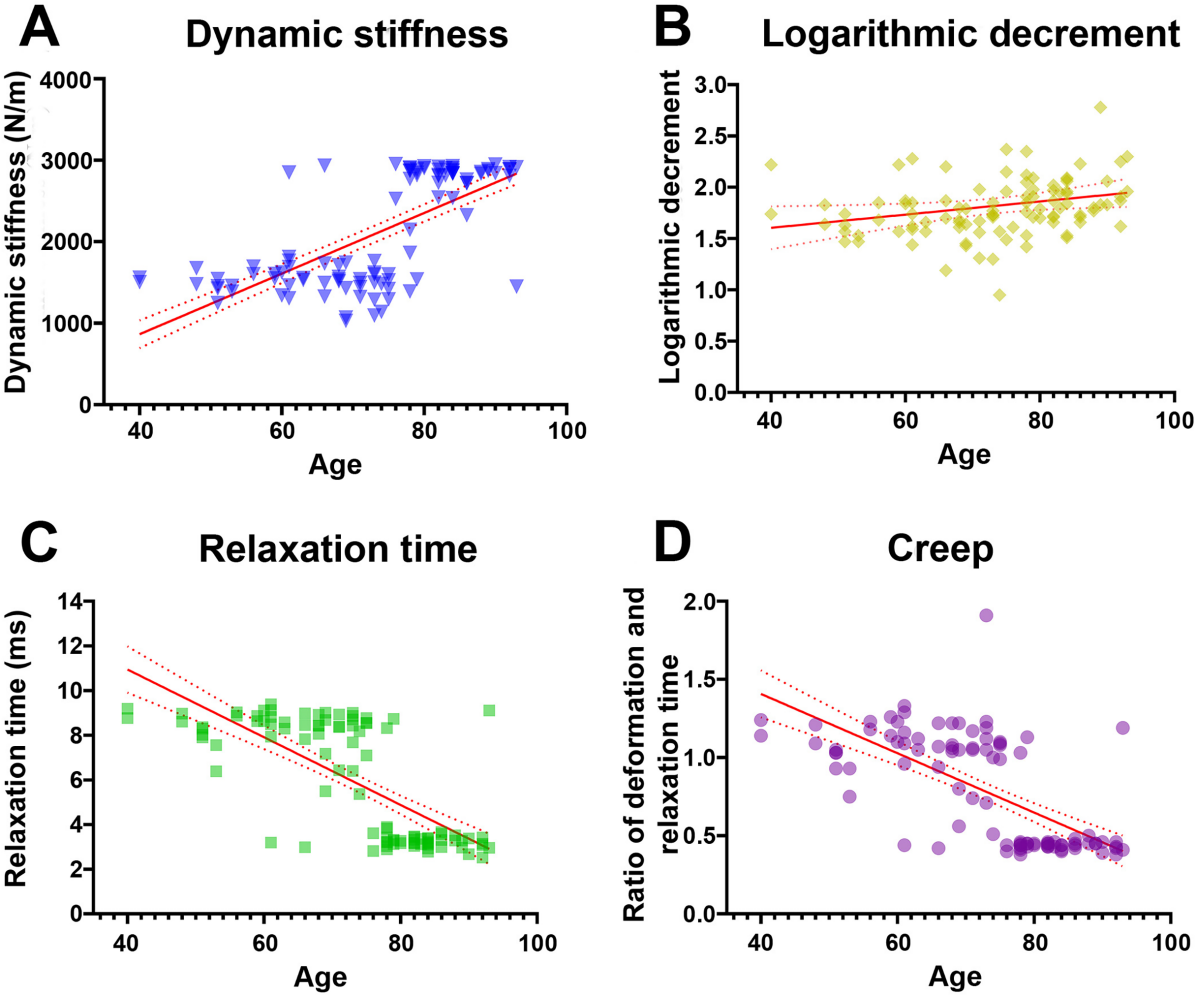
Relationship between age and mechanical properties of transverse carpal ligament (average value from proximal, middle, and distal parts). With advancing age, dynamic stiffness (A) and logarithmic decrement (B) increase while relaxation time (C) and creep (D) decrease.

Pearson’s correlation analysis further confirmed that dynamic stiffness had moderate positive associations (r range 0.65–0.68, *p* < 0.001) with age across all measured locations (Table 3). In terms of elasticity, inferred from the logarithmic decrement, the highest mean values—implying the lowest elasticity—were recorded in individuals aged ≥80 years; however, only distal and average decrement values showed weak but statistically significant correlations with age (r = 0.20 and 0.21, *p* < 0.05). Mechanical stress relaxation time decreased markedly with age, demonstrating moderate to strong negative correlations (r range −0.67 to −0.73, *p* < 0.001), suggesting that stiffer TCLs require less time to rebound to their initial shape once a deforming force is removed. Finally, creep also declined sharply in older specimens, with the ≥80 group displaying significantly lower values and moderate negative correlations with age (r range −0.55 to −0.67, *p* < 0.001), indicative of reduced capacity for gradual elongation under sustained load. Collectively, these findings reveal that the TCL becomes progressively stiffer and less elastic with age, as evidenced by higher dynamic stiffness and logarithmic decrement, alongside lower relaxation time and creep (Fig. 1).

**Table 3:** Correlation of transverse carpal ligament mechanical properties with age.

		Parts of transverse carpal ligament			
		Proximal	Middle	Distal	Average (three parts)
Dynamic stiffness (N/m)	r	0.67	0.65	0.66	0.68
	*p*	<0.001	<0.001	<0.001	<0.001
Logarithmic decrement	r	0.13	0.17	0.20	0.21
	*p*	0.213	0.087	0.043	0.033
Relaxation time (ms)	r	−0.67	−0.73	−0.73	−0.72
	*p*	<0.001	<0.001	< 0.001	<0.001
Creep	r	−0.61	−0.55	−0.65	−0.67
(ratio of deformation and relaxation time)	*p*	<0.001	<0.001	<0.001	<0.001

**Note:**

Pearson’s correlation; *p, p*-value; r, correlation coefficient.

### Histological appearance in the TCL

**Figure 2:**
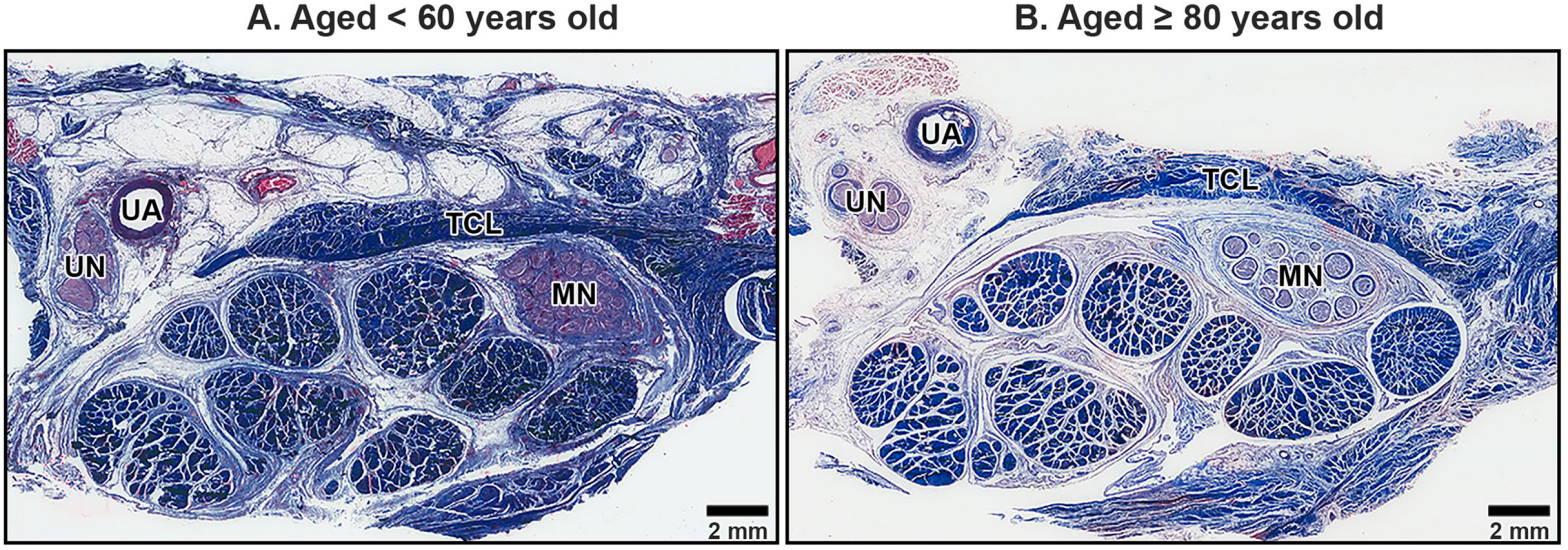
Transverse cross-sections of the right wrist at the level of the middle portion of transverse carpal ligament in cadavers aged <60 years (A) and ≥80 years (B). The transverse carpal ligament (TCL) lies on the top of the carpal tunnel. No apparent difference is seen in the TCL between the under 60-year-old sample and the over 80-year-old sample whereas connective tissue surrounding median nerve (MN) is reduced in aging. Ulnar artery (UA), ulnar nerve (UN), collagen fibers blue, muscle fibers, red blood cell and axon red. Masson’s trichrome staining.

The TCL formed the roof of the carpal tunnel, a flat tubular structure that housed the median nerve and nine extrinsic flexor tendons. These included four tendons of the flexor digitorum superficialis, four tendons of the flexor digitorum profundus, and a single tendon of the flexor pollicis longus. Sub-synovial connective tissue was interspersed among these tendons and the median nerve. At low magnification, there were no readily noticeable differences between younger and older TCL specimens but the obvious alteration in median nerve aging was reduced connective tissue around nerve fiber (Fig. 2).

**Figure 3:**
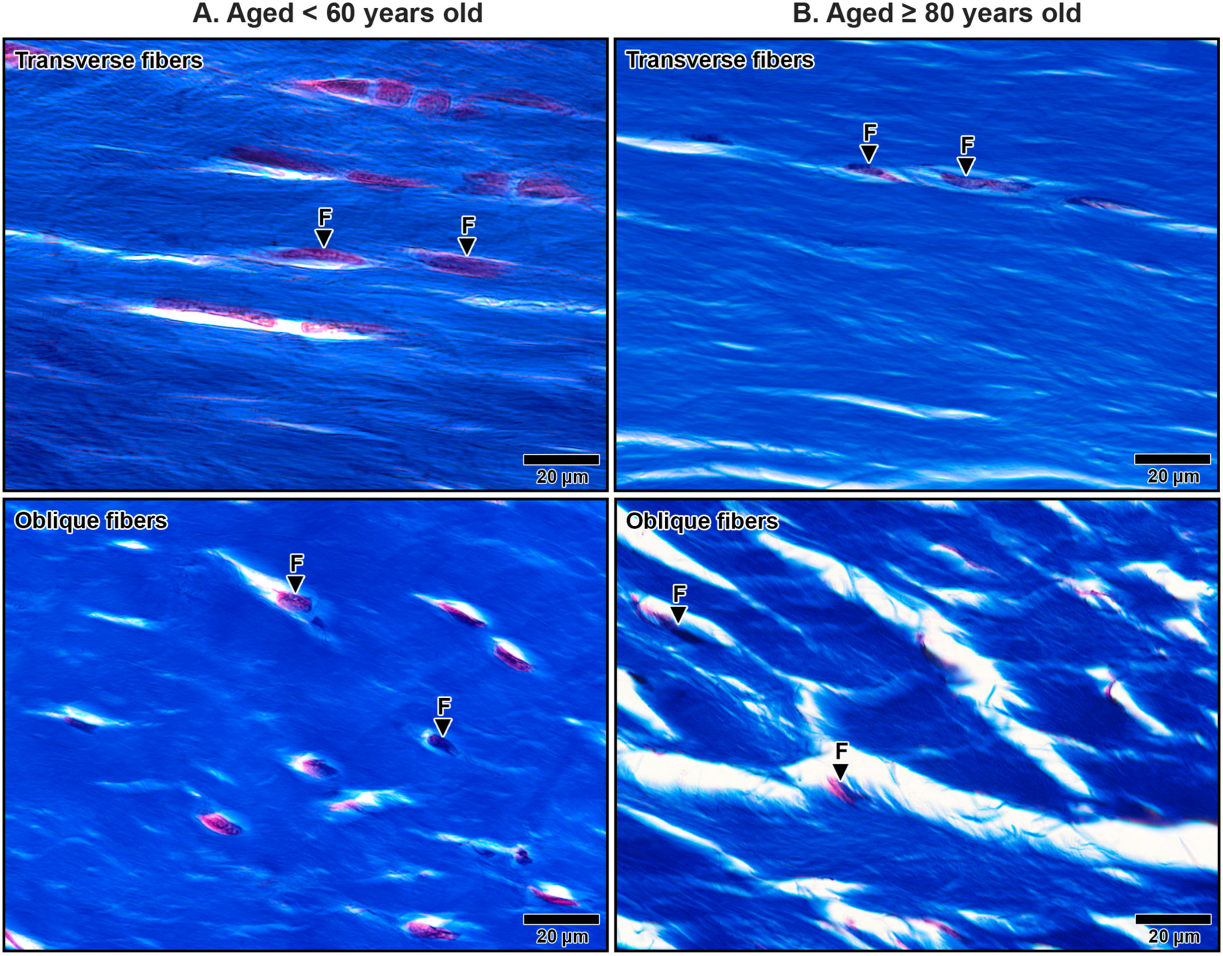
Collagen fiber orientation in the transverse carpal ligament at the middle portion in cadavers aged <60 years (A) and ≥80 years (B). Images show collagen fibers in transverse (top row) and oblique (bottom row) directions. In the under 60-year-old sample, collagen fibers are densely packed and well-organized. In the over 80-year-old sample, collagen fibers appear disorganized with noticeable spacing. Fibroblasts (F) align along the collagen fibers but are reduced in number in the aged sample. Collagen fibers blue, fibroblast nuclei black, fibroblast cytoplasm red. Masson’s trichrome staining.

The primary structural component of the TCL was collagen fibers, which exhibited alignment in both transverse and oblique directions, contributing to the mechanical stability of the carpal tunnel. In transverse sections, collagen bundles appeared either as long, parallel structures or short, oblique segments depending on the orientation of the cut. Spindle-shaped fibroblasts were aligned along the direction of the collagen fibers, contributing to the ligament’s structural organization (Fig. 3).

In specimens from older subjects, the TCL displays pronounced age-related changes. Collagen fibers became less organized, with an increased presence of clefts between collagen bundles, indicating structural degeneration (Fig. 3B). In contrast, younger specimens exhibited well-organized collagen structures with narrow, consistent spacing between bundles (Fig. 3A). Additionally, quantitative analysis revealed a significant 41.9% reduction in fibroblast density in elderly specimens (aged ≥80 years old: 10.68 ± 0.54) compared to that in younger specimens (aged <60 years old: 18.38 ± 0.77, *p* = 0.009), further reflecting the age-related decline in cellularity and matrix integrity.

### Scanning electron microscopic appearance in the TCL

**Figure 4:**
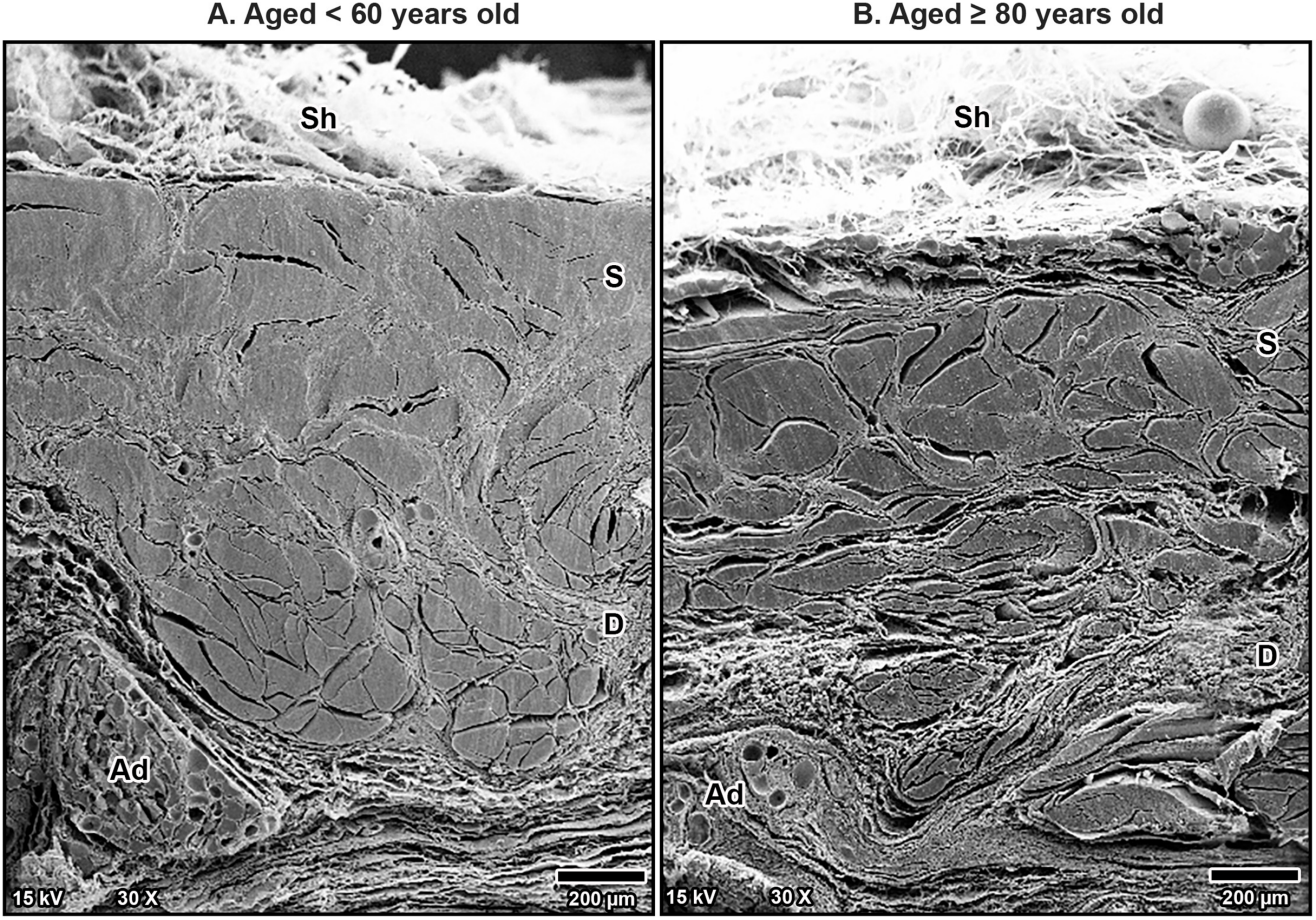
Scanning electron microscopy images of fibrous morphology of the transverse carpal ligament at the middle portion of cadavers aged <60 years (A) and ≥80 years (B). The polygonal collagen bundles are larger in diameter in the superficial part (S) of the transverse carpal ligament and smaller in the deep part (D). In the older specimen, collagen bundles are smaller than that in the younger specimen, especially in the deep layer. Connective tissue sheath (Sh), adipose tissue (Ad).

Scanning electron microscopy revealed that TCL was covered by thin sheath tissue with a loose network of collagen fibers. The collagen fibers in the TCL were organized into tight collagen bundles. These were grouped into fascicles. The collagen bundles were irregular in shape and varied in diameter, with larger bundles predominantly located in the superficial part of the TCL and smaller bundles more common in the deep part (Fig. 4). In older specimens, there was a noticeable increase in the number of small bundles, particularly in the deep portion of the TCL (Fig. 4B), compared to younger specimens, where the bundles were larger and more consistently sized (Fig. 4A).

**Figure 5:**
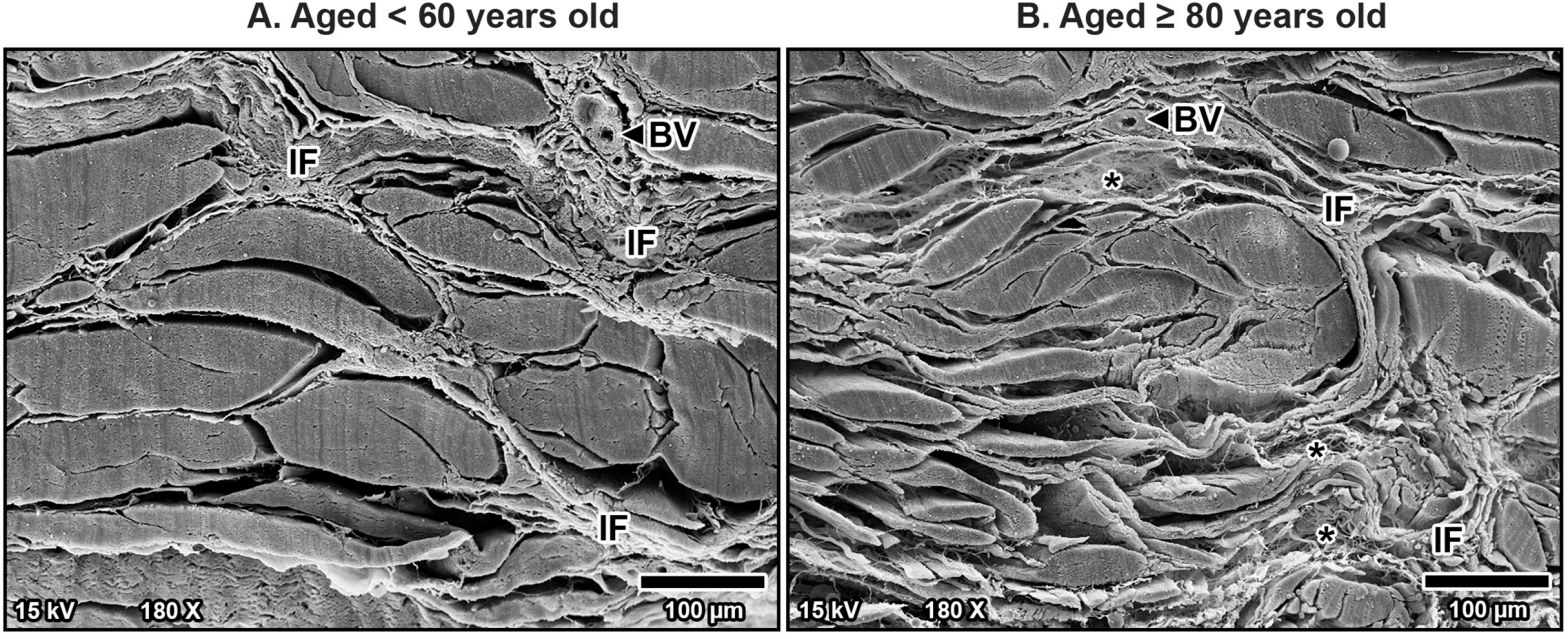
Scanning electron microscopy images of interfascicular matrix morphology in the transverse carpal ligament at the middle portion of cadavers aged <60 years (A) and ≥80 years (B). In the over 80-year-old specimen, the interfascicular matrix (IF) surrounding collagen-rich fascicles undergoes disorganization with fine collagen meshwork (asterisks) compared to well-arranged parallel collagen in the interfascicular matrix of younger specimen. Blood vessels (BV).

Additionally, fine collagen fibers were more prominently observed in the interfascicular matrix of the aging TCL. This matrix, a connective tissue surrounding the fascicles, became denser with more visible fine collagen fibers as a mesh-like pattern in the TCL in older specimens (Fig. 5B). By contrast, younger specimens displayed well-arranged collagen in parallel manner in the interfascicular matrix, resulting in a more distinct separation of fascicles (Fig. 5A). The presence of blood vessels within the interfascicular matrix was also evident and more pronounced in the young TCL.

**Figure 6:**
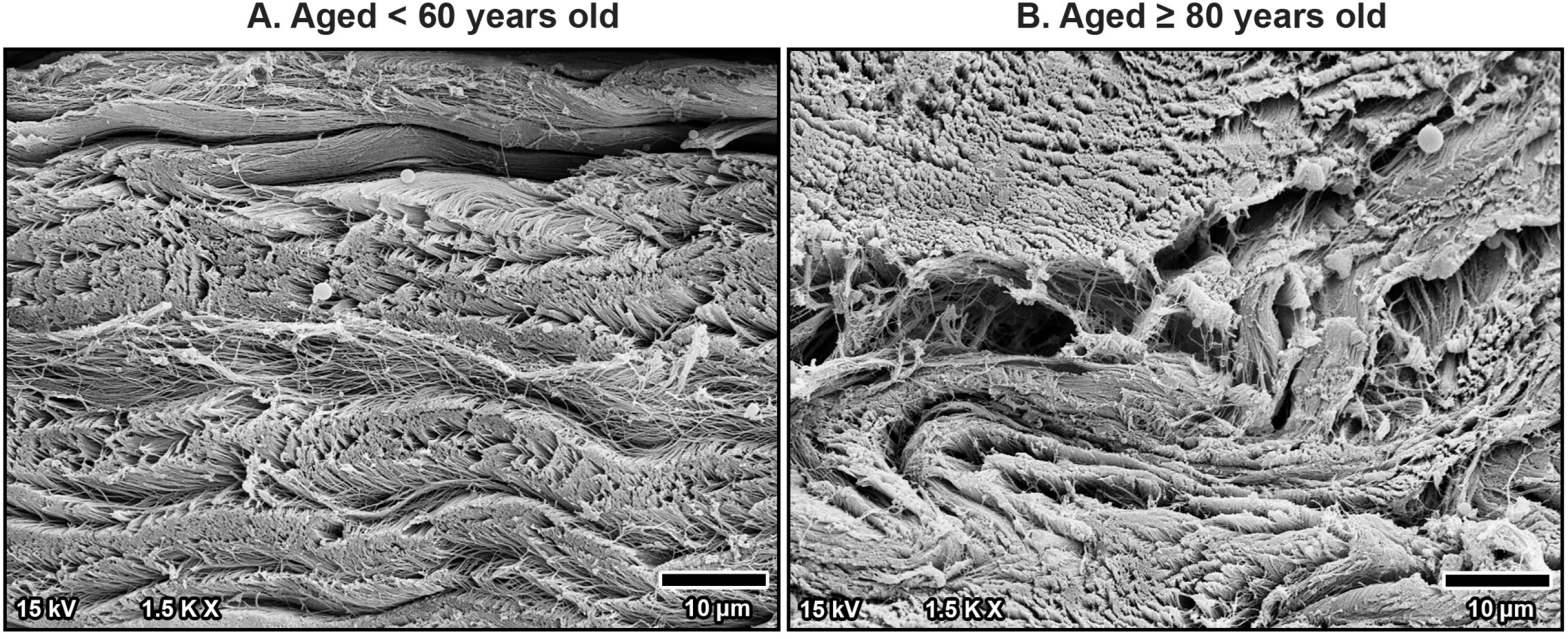
High magnification scanning electron microscopy images of fibrous morphology in the transverse carpal ligament at the middle portion from cadavers aged <60 years (A) and ≥80 years (B). The under 60-year-old transverse carpal ligament shows regularly arranged and tightly packed collagen fibers. In contrast, the over 80-year-old specimen displays disorganized collagen fibers with irregular spacing and fragmentation.

At higher magnification, collagen fibers within the bundles were well-arranged and tightly packed in younger specimens, maintaining a regular organization (Fig. 6A). However, in older specimens, the collagen bundles were irregularly arranged and showed signs of disorganization and fragmentation, indicating significant age-related structural deterioration (Fig. 6B). These age-associated changes in the collagen arrangement and organization contribute to the stiffening and loss of flexibility in the TCL.

### Histological appearance in the median nerve under TCL

**Figure 7:**
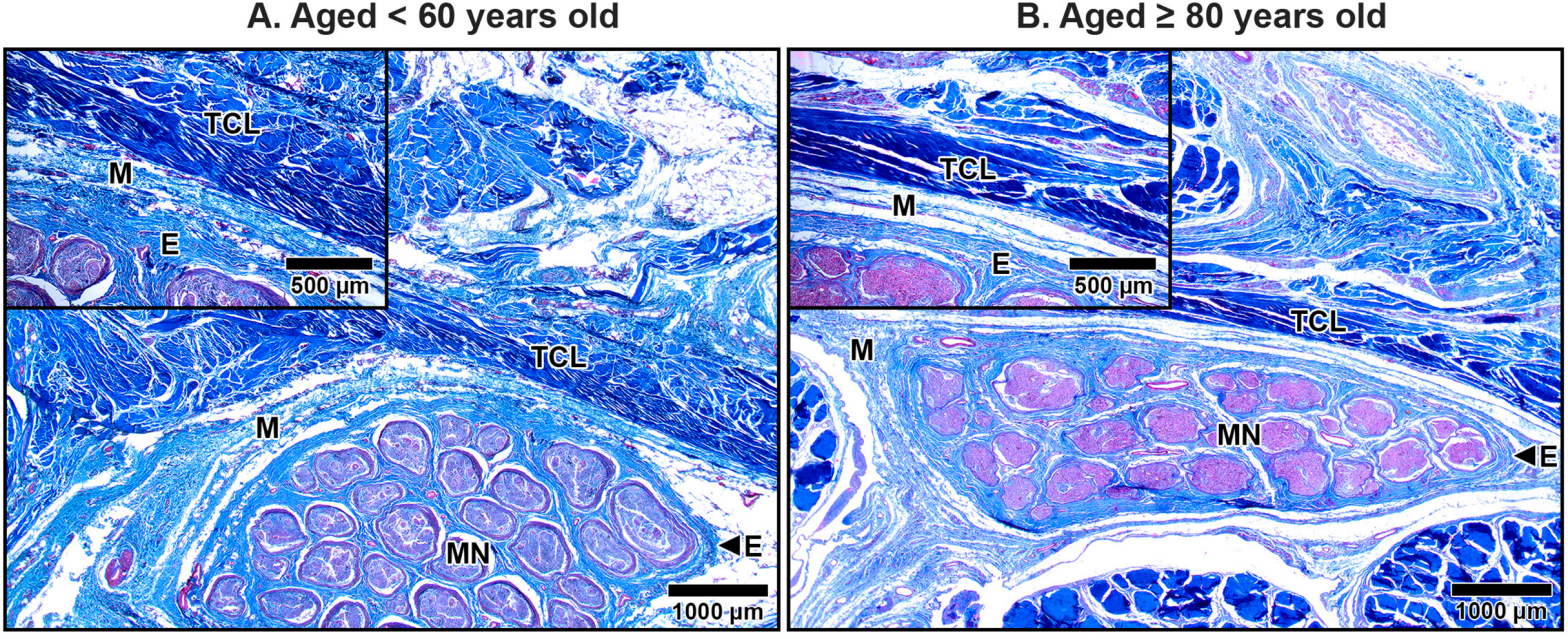
Median nerve beneath the middle portion of the transverse carpal ligament in cadavers aged <60 years (A) and ≥80 years (B). In the under 60-year-old subject, the mesoneurium (M) and epineurium (E) around the median nerve (MN) appear thick and rich in collagen fibers. In contrast, the over 80-year-old shows reduced thickness and density of the mesoneurium and epineurium, reflecting age-related degeneration. Inset images show median nerve-transverse carpal ligament distance. The median nerve is closer to transverse carpal ligament (TCL) in the older specimen than in younger specimen due to thinning of the mesoneurium and epineurium. Masson’s trichrome staining.

**Figure 8:**
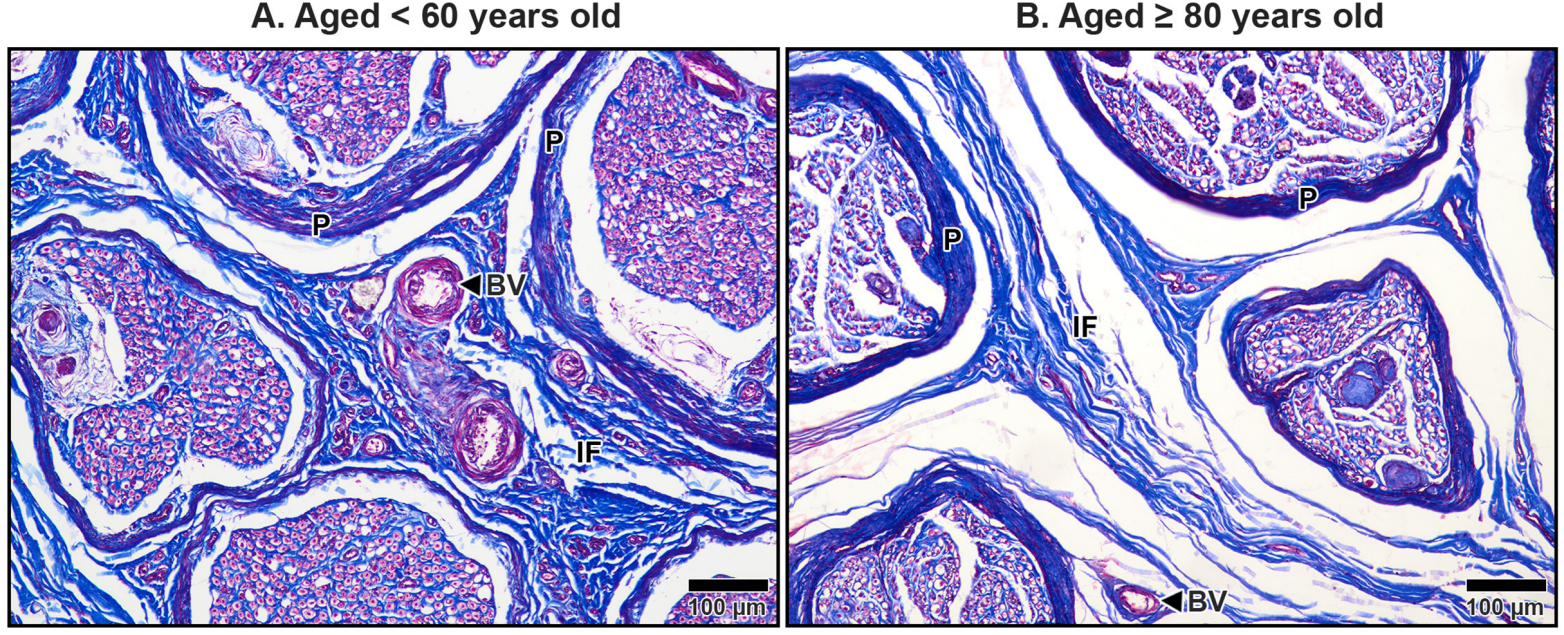
Interfascicular epineurium of the median nerve beneath the middle portion of the transverse carpal ligament in cadavers aged <60 years (A) and ≥80 years (B). Dense and well-organized collagen fibers are visible in the interfascicular epineurium (IF) of the under 60-year-old. In contrast, the over 80–year-old shows loosely packed and disorganized collagen in the interfascicular tissue with larger spaces. Blood vessels (BV), perineurium (P). Masson’s trichrome staining.

**Figure 9:**
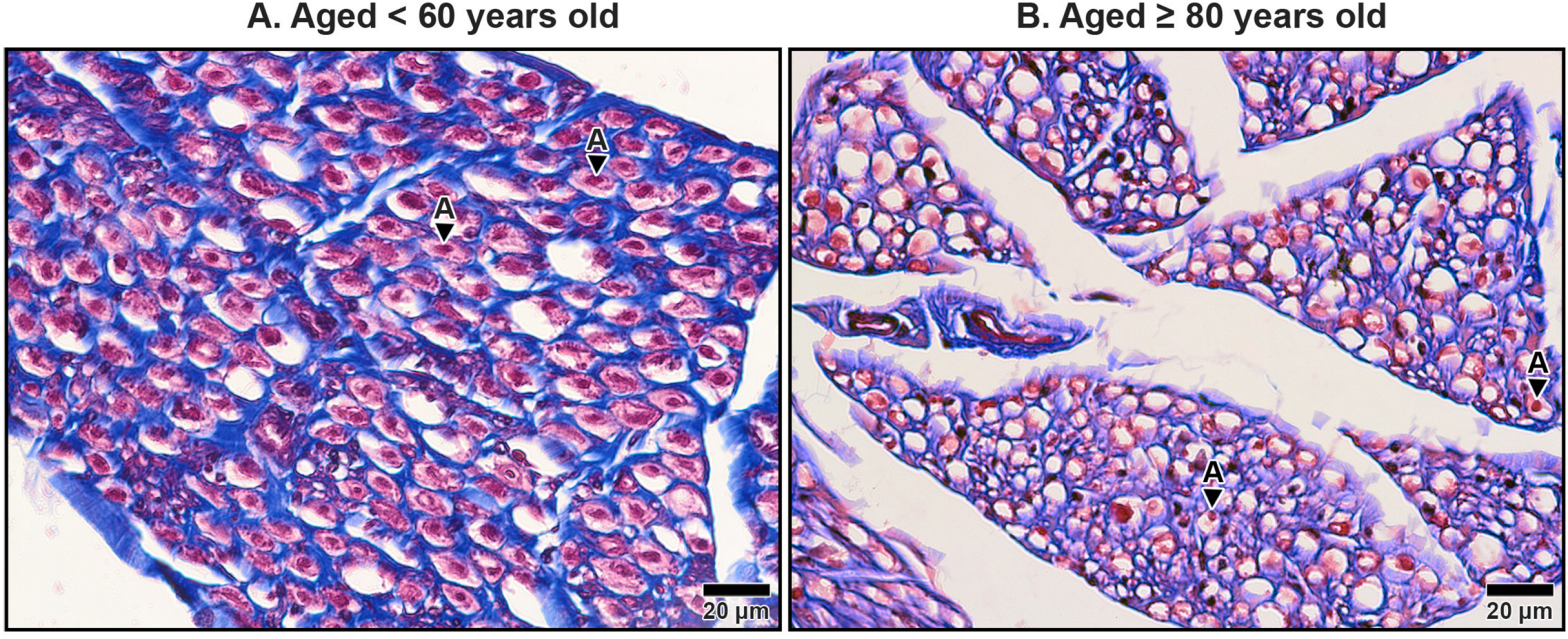
Endoneurium and myelin sheath of axons in the median nerve beneath the middle portion of the transverse carpal ligament in cadavers aged <60 years (A) and ≥80 years (B). In the under 60-year-old specimen, thick endoneurium (blue ring) and well-preserved myelin sheaths (pale red areas surrounding axons) are observed, along with densely packed axons (A). In the over 80-year-old specimen, the endoneurium is thinner and disorganized. Axonal atrophy and axon loss are apparent in the older sample together with reduced, fragmented and lost myelin sheaths, indicating significant age-related degeneration. Masson’s trichrome staining.

The median nerve consists of multiple fascicles, each enclosing numerous axons. Each axon was surrounded by a thin connective tissue layer known as the endoneurium, while each fascicle was encapsulated by a denser connective tissue sheath called the perineurium. Surrounding all fascicles was the epineurium, a thicker sheath composed primarily of collagen fibers, which provided structural integrity to the nerve. Beyond the epineurium lay the mesoneurium, the outermost connective tissue layer that anchored the nerve to surrounding structures. The mesoneurium, epineurium, and interfascicular epineurium also contained blood vessels that supplied the nerve and its associated structures, which became progressively smaller as they penetrated deeper, transitioning into capillaries within the endoneurium (Figs. 7–9).

In aging specimens, significant structural changes were observed in these connective tissue layers. The mesoneurium and epineurium showed a reduction in thickness and collagen density in older subjects compared to younger ones (Fig. 7). This reduction narrowed the distance between the TCL and the median nerve (Fig. 7B), which may impair the nerve’s ability to expand and contract during wrist and hand movements, potentially exacerbating nerve compression under the TCL.

The interfascicular epineurium in older specimens exhibited a decrease in collagen fiber density and increased loosening, as evident in Fig. 8B. By contrast, younger specimens maintained a more compact and well-organized collagen matrix within the interfascicular epineurium (Fig. 8A). Similarly, the endoneurium surrounding individual axons showed reduced collagen density in older specimens, reflecting an age-related deterioration of this structural layer (Fig. 9B).

The myelinated fibers in the median nerve also underwent significant age-related degeneration. In younger specimens, the myelin sheaths surrounding axons were thick, continuous, and abundant (Fig. 9A). In contrast, older specimens exhibited reduced frequency and thinner, fragmented myelin sheaths. Additionally, degenerative changes were noted in the axons themselves, with evidence of axonal atrophy and a substantial loss of axons in older subjects (Fig. 9B). These findings highlight the cumulative impact of aging on the structural integrity of the median nerve, which may contribute to functional impairments and increased susceptibility to conditions such as CTS.

## Discussion

This study aimed to investigate age-related alterations in the TCL and the median nerve, focusing on biomechanical properties, histological changes, and their potential contributions to the increased prevalence and severity of CTS in older adults. Three main findings emerged: (1) a significant correlation between age and changes in TCL biomechanical properties, including increased stiffness and reduced elasticity, relaxation time, and creep; (2) progressive structural disorganization of the TCL, characterized by reduced fibroblast density, disorganized collagen fibers, and an altered interfascicular matrix; and (3) significant degenerative changes in the median nerve, including thinning of connective tissue sheaths, axonal atrophy, and myelin sheath loss in older specimens. Together, these findings highlight the structural and functional vulnerabilities in the carpal tunnel that may underlie the greater susceptibility of elderly individuals to CTS.

Aging was associated with substantial changes in the mechanical properties of the TCL. Stiffness showed a strong positive correlation with age, indicating a progressive increase in the ligament’s rigidity. Conversely, elasticity, mechanical stress relaxation time, and creep showed significant negative correlations with age, suggesting diminished capacity for stretch, deformation, and recovery. These changes likely reflect a cumulative loss of adaptability in the TCL’s extracellular matrix over time, impairing its biomechanical buffering capacity during repetitive wrist and hand movements. As the ligament stiffens, it exerts greater compressive forces on the median nerve, contributing to entrapment neuropathy and the clinical manifestations of CTS. Furthermore, an increased stiffness in TCL could directly impact on the surgical procedure, potentially a prolonged operative duration and the subsequent recovery.

Our findings align with prior studies on age-related changes in other connective tissues. Previous studies reported a similar increase in stiffness with age in the medial collateral ligament, particularly in older adults (Aronson et al., 2014) as well as increased stiffness in the plantar fascia of individuals over 65 years (Çevik Saldıran, Kara & Öztürk, 2023). However, a decrease in stiffness with age in the anterior cruciate ligament (ACL) of the knee was reported (Woo et al., 1991), a discrepancy that may be attributed to differences between intra-articular ligaments such as the ACL, which are exposed to synovial fluid, and extra-articular ligaments such as the TCL. Synovial exposure may promote enzymatic degradation in intra-articular ligaments, leading to softer tissue in older specimens (Ishibashi et al., 2022).

Histological analysis revealed that fibroblast density in the TCL declines significantly with age. Fibroblasts are critical for maintaining ligament homeostasis, as they synthesize and remodel collagen and other extracellular matrix components. The observed reduction in fibroblasts is consistent with studies in other ligaments, such as the ACL (Hasegawa et al., 2013) and periodontal ligament (Krieger, Hornikel & Wehrbein, 2013), where fibroblast density diminishes with advancing age. This decline may be driven by telomere shortening and mitochondrial dysfunction, which lead to cellular senescence and apoptosis (Bernadotte, Mikhelson & Spivak, 2016; Maldonado et al., 2023; Rossiello et al., 2022). The loss of fibroblasts likely reduces the TCL’s reparative capacity, exacerbating structural deterioration and contributing to its mechanical stiffening.

Age-related disorganization of collagen fibers was another prominent finding. Fine collagen fibrils became fragmented and misaligned, with larger interfibrillar spaces, particularly in the aging TCL. This is consistent with increased matrix metalloproteinase (MMP) activity during aging, which degrades collagen and disrupts fibrillar organization (Fisher, Varani & Voorhees, 2008). Additionally, advanced glycation end-products formed through non-enzymatic cross-linking of collagen may stiffen the extracellular matrix, further reducing its flexibility and increasing tissue rigidity (Bailey, Paul & Knott, 1998; Sloseris & Forde, 2024). These structural changes are compounded by reductions in water content and lubrication within the extracellular matrix, which are known to occur in aging ligaments (Osakabe et al., 2001; Tohno et al., 1999). The cumulative effect of these changes renders the TCL less capable of withstanding mechanical stress, increasing its potential to compress the underlying median nerve.

Degenerative changes in the median nerve were striking and paralleled those observed in the TCL. The thinning of the mesoneurium, epineurium, perineurium, and endoneurium in older specimens represents a significant loss of protective connective tissue layers. These findings align with previous studies that observed a reduction in collagen content within the nerve sheaths of aging rats (Bahcelioglu et al., 2008) as well as thinning of the sciatic nerve (Esquisatto et al., 2014). The loss of these connective tissue layers compromises the structural integrity of the median nerve, making it more susceptible to compression by the stiffened TCL. Additionally, thinner nerve sheaths may impair the nerve’s ability to maintain internal pressure and resist external tensile forces, further increasing its vulnerability to mechanical stress (Ju, Lin & Chang, 2017).

Axonal and myelin sheath degeneration in the aging median nerve further emphasize the deleterious effects of aging on nerve function. Axonal atrophy, reduced myelin density, and fragmentation observed in this study are consistent with prior research on the human sural nerve (Jacobs & Love, 1985) and animal models (Ceballos et al., 1999; Chase et al., 1992; Verdú et al., 2000). Mechanistically, aging is associated with impaired axonal transport, reduced expression of myelin proteins, and mitochondrial dysfunction, which collectively contribute to axonal degeneration and myelin breakdown (Melcangi et al., 1998; Xing et al., 2012). These changes may explain the slower motor and sensory conduction velocities reported in older adults, as the loss of myelin and axonal integrity impairs signal transmission (Werner et al., 2012). In the context of CTS, these age-related nerve changes may exacerbate the functional impact of median nerve compression, leading to more severe clinical symptoms in older patients.

### Clinical implications

The findings of this study have important implications for understanding the pathophysiology of CTS in older adults. The age-related stiffening and disorganization of the TCL, combined with structural degeneration of the median nerve, creates a biomechanical and neurophysiological environment that predisposes elderly individuals to CTS. This may explain why CTS is not only more prevalent in older adults but also more severe, with higher rates of functional impairment and less satisfactory outcomes following surgical intervention (Fung, Tang & Fung, 2015; Vyšata et al., 2014). Recognizing these age-related changes underscores the need for preventive and therapeutic strategies tailored to the elderly population. For example, physical therapies aimed at maintaining TCL flexibility or pharmacological approaches targeting MMP activity and glycation may help mitigate these structural changes.

### Study limitations and future directions

This study is not without limitations. The use of formalin-embalmed cadavers may alter the mechanical properties of the TCL, potentially affecting the generalizability of the findings to living tissues. This study did not evaluate the role of proteoglycans or ground substances, which are integral components of the extracellular matrix and may significantly influence ligament mechanics. Additionally, the absence of detailed medical histories may represent a potential confounding factor, as systemic diseases could influence ligament and nerve properties. This study focused on proximal–distal variations of the TCL overlying the median nerve; mediolateral thickness and detailed nerve pathway orientation were not evaluated and warrant further investigation. The unequal distribution of specimens across age groups, particularly the smaller number of younger cadavers, reflects the inherent nature of cadaveric studies and may introduce bias favoring older age groups. Future research also should investigate these components and explore therapeutic interventions that could slow or reverse age-related changes in the TCL and median nerve. Furthermore, *in vivo* studies using advanced imaging and biomechanical modeling could provide a more comprehensive understanding of carpal tunnel biomechanics in aging populations.

## Conclusions

This study highlights significant age-related changes in the TCL and median nerve that contribute to the pathophysiology of CTS in older adults. Structural changes in the TCL, including increased stiffness, fibroblast loss, and collagen disorganization, were accompanied by degeneration of the median nerve, including thinning of connective tissue sheaths, axonal degeneration, and myelin breakdown. These findings provide valuable insights into the mechanisms underlying the higher prevalence and severity of CTS in the elderly, emphasizing the need for targeted interventions to address these age-associated changes.

## Supplemental Information

10.7717/peerj.20878/supp-1 Supplemental Information 1 Raw data of all experiments.

## Additional Information and Declarations

### Data Availability

The following information was supplied regarding data availability:

The raw data is available in the Supplemental File.
